# Quasi-Static Compression Properties of Bamboo and PVC Tube Reinforced Polymer Foam Structures

**DOI:** 10.3390/polym13203603

**Published:** 2021-10-19

**Authors:** J. J. N. Amelia, M. Y. M. Zuhri, Z. Leman, N. I. Zahari, A. As’arry, R. A. Ilyas

**Affiliations:** 1 Advanced Engineering Materials and Composites Research Centre, Department of Mechanical and Manufacturing Engineering, Faculty of Engineering, Universiti Putra Malaysia, Serdang 43400, Malaysia; ameliajuria96@gmail.com (J.J.N.A.); zleman@upm.edu.my (Z.L.); rubie@upm.edu.my (N.I.Z.); 2 Laboratory of Biocomposite Technology, Institute of Tropical Forestry and Forest Product (INTROP), Universiti Putra Malaysia, Serdang 43400, Malaysia; 3 Department of Mechanical and Manufacturing Engineering, Faculty of Engineering, Universiti Putra Malaysia, Serdang 43400, Malaysia; zizan@upm.edu.my; 4 School of Chemical and Energy Engineering, Faculty of Engineering, Universiti Teknologi Malaysia, UTM, Johor Bahru 81310, Malaysia; ahmadilyas@utm.my; 5 Centre for Advanced Composite Materials (CACM), Universiti Teknologi Malaysia, UTM, Johor Bahru 81310, Malaysia

**Keywords:** compression strength, foam, specific energy absorption, tube

## Abstract

In recent years, there has been a growing interest for composite materials due to the superior capability to absorb energy and lightweight factor. These properties are compatible to be utilized in the development for transportation system as it can reduce the fuel consumption and also minimize the effect of crash to the passenger. Therefore, the aim for this project is to study the compression strength and energy absorbing capability for Polyvinyl chloride (PVC) and bamboo tubes reinforced with foam. Several parameters are being considered, these being the effect of single and multiple tube reinforced foam structure, foam density, diameter of the tube as well as effect of different crosshead speed. The results showed that increasing the relative foam density will led to an increase in the compression strength and specific energy absorption (SEA) values. Furthermore, a significant increase of compression strength can be seen when several tubes are introduced into the foam while SEA remained almost the same. Finally, the influence of crosshead below 20 mm/min did not vary significantly for both compression strength and SEA.

## 1. Introduction

An energy absorbent structure can be a structure that exchanges half or all of the mechanical energy into another sort of energy. The energy recover will either be reversible similarly in the case of elastic strain energy in solids or it will be irreversible like plastic dissemination of energy related with lasting distortion of the strong structure. Energy absorbing structure design and analysis differ significantly from standard structural design and analysis. Energy absorbing structures must withstand high impact loads due to the complicated deformation and failure by significant geometry changes, strain hardening effects, strain-rate effects, and interactions between distinct deformation modes including bending and stretching [1]. There are several types of sandwich cores that have been studied in recent years. The determination for the core material and its design are crucial and might be vary depending on the application. In addition, structural elements are inserted into the core structures such as foams and honeycombs to modify mechanical properties of the sandwich structures such as core compression strength, buckling instability and in-plane shear [2]. In typical structural applications, the thickness of the face sheet rarely exceeds several millimetres, whereas the thickness of core can be over 50 mm [3]. In a study by Colloca et al. [4] they have reported that the compressive modulus of the foam (Divinycell PVC) increases as the relative density of the foam increases, but the densification strain decreases. Also, a comparison of absorbed energy shows that the energy absorption during compression process rise up to 600% more than under tension due to greater strain value.

To develop crashworthy structures, tubular structures have been commonly used in the design of tube reinforced foam structure. The most frequent and oldest type of foldable energy absorber is thin tubes. When a thin-walled steel tube is subjected to an axial compressive load, it can generate either a concertina (axisymmetric buckle) or a diamond (non-axisymmetric buckle). A concertina deformation mode is most efficient energy absorbing mode [5]. Khan et al. [6] explained that failure mode for thin wall cylindrical composite tube can be divided into three modes; (1) mushrooming failure, (2) brittle fracture of the composite tube and (3) increasing folding and hinging. In addition, the energy absorbing capacity is highest at the first mode, medium at third mode and the least at second mode. Zuhri et al. [7] examined the energy absorbing properties of bamboo-based structure by conducting quasi-static and dynamic tests. According to the data, the energy absorbing capability of individual bamboo tubes increases when the diameter-to-thickness ratio (D/t) decreases. Also, the small diameter of bamboo tubes showed more noticeable crushing than the larger size. A study from Zhou et al. [8] suggested that by embedding tubes in a foam panel, it will influence the failure process within the composite tubes by significantly improving their ability to absorb energy. However, the SEA values of the hybrid tube reinforced foams were found to be insensitive to variations in foam density. It is important to note that tube-based foams have a larger energy-absorbing capacity than many comparable core systems, emphasising its potential for usage in extreme crushing situations. In a study conducted by Alhawamdeh et al. [9] shows that the failure mode of the axially loaded hollow pultruded fibre-reinforced polymer (PFRP) profiles varied depending on their cross-sectional shape. The hollow box profiles are dominated by local buckling of the walls, whereas the hollow circular profiles dominated by compressive and shear failure at the profiles ends. The results stated that the hollow circular PFRP profiles, the failure mode is the same for all length-to-width (L/D) ratios.

This paper introduces a new combination structure for the purpose to enhance an energy absorption of the current foam structure. Attention is focused on understanding the energy absorbing characteristics of single and multiple tubes reinforced foam structure under quasi-static loading conditions.

## 2. Materials and Methods

### 2.1. Material

Two types of crosslinked PVC foams with different densities are used in this study, which are Divinycell F50 and Divinycell HP80 foams with a density of 50 kg/m^3^ and 80 kg/m^3^, respectively. The foams are supplied by DiabGroup and comes in the shape of flat panel, which are color-coded to differentiate the foam type and both are having thickness of 20 mm. The selected tubes are Polyvinyl chloride (PVC) and eco-friendly bamboo tube. The commercial PVC tubes used are round conduit where it is mainly used for construction, electrical, etc. Two different sizes of tubing are selected, where the outer diameters (D_o_) are 20 mm and 25 mm. Then, it is cut into 20 mm length to ensure it having the same height as the foam. The length of the specimen is similar to work conducted by Alia et al. [10] and Cinar [2]. On the other hand, bamboo tubes used are readily available in the laboratory and it is light brown in colour. This bamboo is originally used to create a beautiful garden edging and is chosen due to their highly versatile natural resource that is easily to sustained and eco-friendly. The selection of D_o_ for the bamboo tube is based on diameter size that is close to 25 mm, due to it is not uniform in nature. Prior to testing, the tubes are cut into 20 mm length (similar reason as for PVC tube) using the circular saw from the internode parts of the bamboo. It is known that the diameter of the node part is slightly larger than the internode part and can affect the structural performance, as investigated by Molari et al. [11]. However, in this study, the node is not considered to ensure the insertion tube having an interference fit to the foam. The physical properties of the tested tubes are shown in Table 1.

### 2.2. Fabrication and Mechanical Testing

**Figure 1:**
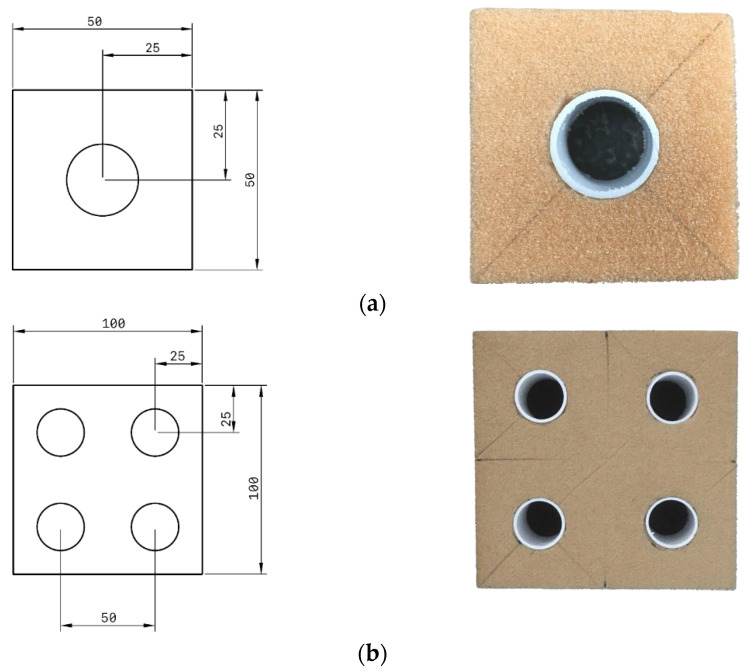
Dimension and positioning for (**a**) single tube and (**b**) multiple tube reinforced foam structure.

Initially, foam thickness of 20 mm is cut into block of square with a dimension of 50 × 50 mm. Then, a hole is formed using circular drill at the centre of the foam to allow an identical size of tube to be inserted. Single tube-reinforced foam samples are designed by embedding a 20 mm long PVC and bamboo tubes into the Divinycell F50 and HP80 foam. In addition, for multiple tubes, the size of the square block is double than the single tube and using the same method as for single tube. Figure 1 shows the illustration and sample of the structure (the unit is in mm).

**Figure 2:**
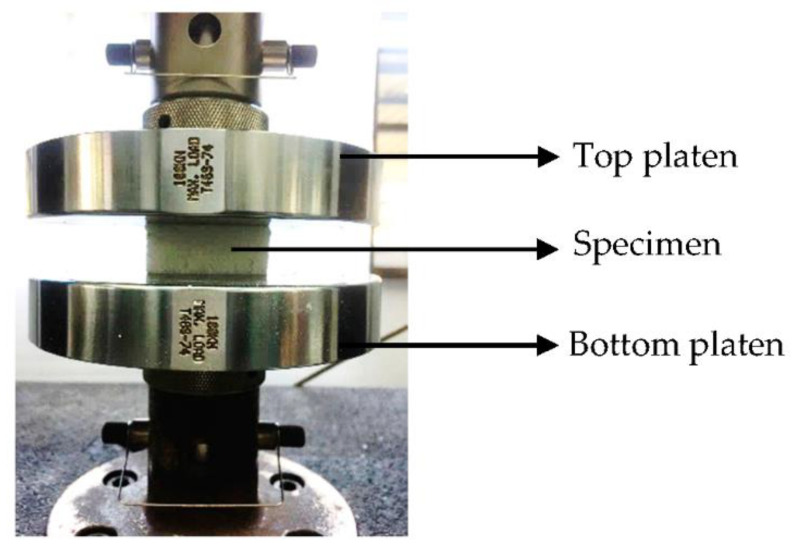
Quasi-static compression testing of specimen.

Later, a series of axial quasi-static compression testing is performed using Universal Testing Machine Instron model 3382 with a load cell of 100 kN as shown in Figure 2. Each of the specimens are tested at a constant crosshead speed of 2 mm/min. For this purpose, compression test is carried out in accordance to ASTM C365 [12]. For each of the test configurations, three specimens are replicated.

The specimen is axially crushed between the parallel steel flat platens by placing it on the bottom platen. The crosshead is then lowered until the specimen come into touched with the surface of the top platen. The quasi-static tests are continued until it reaches a compaction point where the curve begins to rise up steeply after completing the sustained crushing [13]. For analysis purposes, the mechanism of failures is monitored, and the deformation process images are taken throughout the tests. The load-displacement raw data is used to determine the compression strength and specific energy absorption characteristics of the structures. Finally, the multiple tubes reinforced foam structures also being tested using different crosshead speed of 5 and 20 mm/min. Prior to testing the specimens, each specimen is labelled with a code for easily identification. Table 2 shows the code used for single and multiple tube reinforced foam structures.

## 3. Results and Discussion

### 3.1. Compressive Behaviour of Foam Material

**Figure 3:**
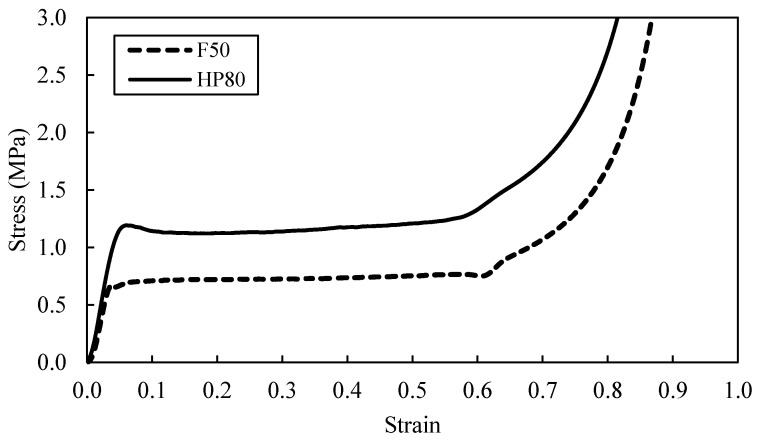
Stress-strain curves of F50 and HP80 foam.

A typical stress-strain curves following quasi-static test on the F50 and HP80 foam is shown in Figure 3. There are three phases during the compression process. Initially, in the elastic regime, the material response is roughly linearly occurred up to approximately 4% strain and near to the yield point where the elastic response ends. This is following the Hooke’s law, which stated that the strain is proportionate to the applied stress. Next, at the beginning of the crushing regime, a constant plateau stress is forming after the first substantial deviation from the linear regime. Finally, the densification regime begins where the force increases drastically with little deformation. This illustrates on how the foam materials have unique properties such as the ability to deform extensively while sustaining modest amounts of stress before reaching the densification regime [1,14].

Finally, the plateau region gradually ends as stiffening occurs when the cell walls collapse and started to interact with the neighbouring cell walls of the foam. This continuous interaction condition results in a rapidly increasing strain where the densification take place at 70 to 80% strain. It is also can be seen that an increase in density will also increase the compressive strength and the SEA. For example, an increase in density from 50 to 80 kg/m^3^ resulted in 71% increase in the compressive strength. The values obtained from the experimental testing is summarised in Table 3.

### 3.2. Compression Behaviour of Single Tube

**Figure 4:**
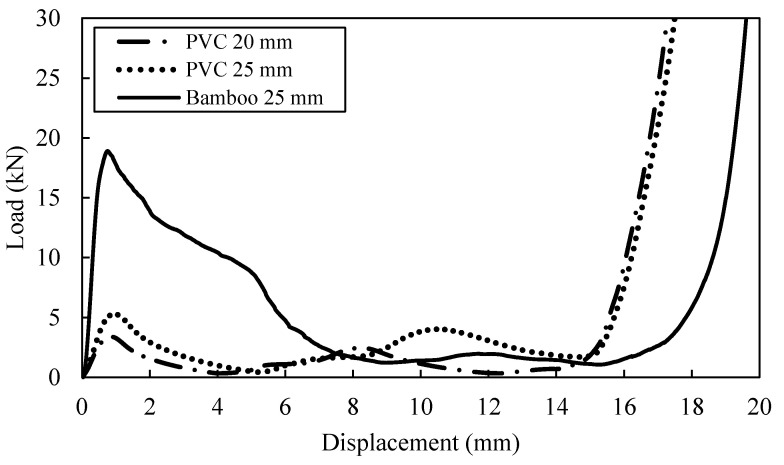
Load-displacement curves for three types of tube.

**Figure 5:**
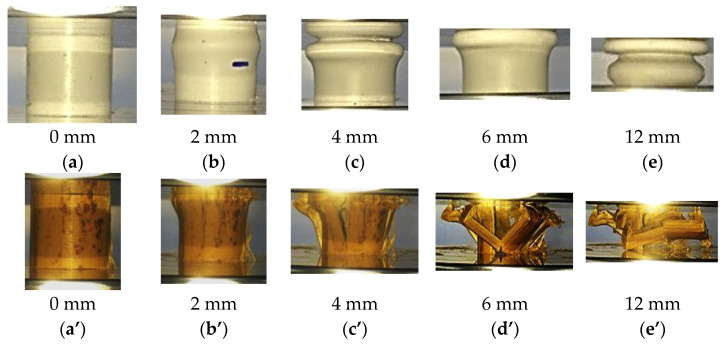
The compression modes at several displacement values of (**a**)–(**e**) PVC tube and (**a’**)–(**e’**) Bamboo tube.

In Figure 4, the load-displacement curves present the behaviour of PVC and bamboo tubes. As for PVC tubes, identical traces of compressive force applied is observed, where the force rose until it reached a point before the tube wall buckled and formed a fold due to interpenetration collapsed. The development of the first fold in the PVC tube occurred at the same time that the initial peak force is obtained. Following this, the two sections of formation plastic hinges at cell wall junctions are compacted together while folding process continued to occur. Compaction of the PVC tube would typically cause to increase the load as the curve displayed a second peak. However, folding process will weaken the PVC tubes which causes the load to drop after second peak until the tube is fully compacted as shown in Figure 5a,b. Khan et al. [6] suggested that compaction and delamination can be happened at the same time, where they balanced each other out which yielding in a relatively sustained crushed until reached the densification point.

**Figure 6:**
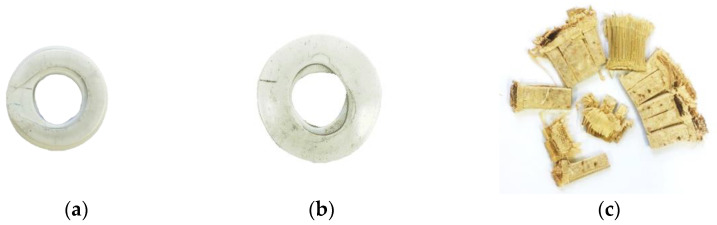
Shape of tube after deformation: (**a**) PVC Ø 20 (**b**) PVC Ø 25 and (**c**) Bamboo.

Similarly for the individual bamboo tube, during the crushing process of the bamboo tube, the tube wall did not fail as in PVC tube, this is due to the bamboo has higher stiffness compared to PVC. The longitudinal fractures of bamboo developed when the load exerted on the tube resulting in tube wall separated into several part as shown in Figure 5a’–e’ or Figure 6c. Thus, this event further clarifies why the second peak did not occur for bamboo tube as in the case with the PVC tube.

In comparison, the individual 25 mm diameter of PVC tube (D/t = 12.5) offer greater compression strength compared to the 20 mm of PVC tube (D/t = 13.4) counterpart. It is noted that the SEA value for 20 mm PVC tube is 7.74 kJ/kg which is less than 25 mm PVC tube, that is 9.58 kJ/kg. With the increasing of D/t ratio, lower value in compressive strength and the specific energy absorption have been recorded during the testing process. In addition, the SEA value for bamboo tube (D/t = 4.82) is approximately 21.95 kJ/kg, which is greater than both PVC tubes due to the lower diameter-to-thickness ratio as suggested by [7]. In terms of compressive strength and modulus, the bamboo tube dominates the response of axial compression loading as shown in Figure 4. The failure deformation of the PVC and bamboo tubes are photographed in Figure 5 and Figure 6.

### 3.3. Single Tube Reinforced Foam Structures

**Figure 7:**
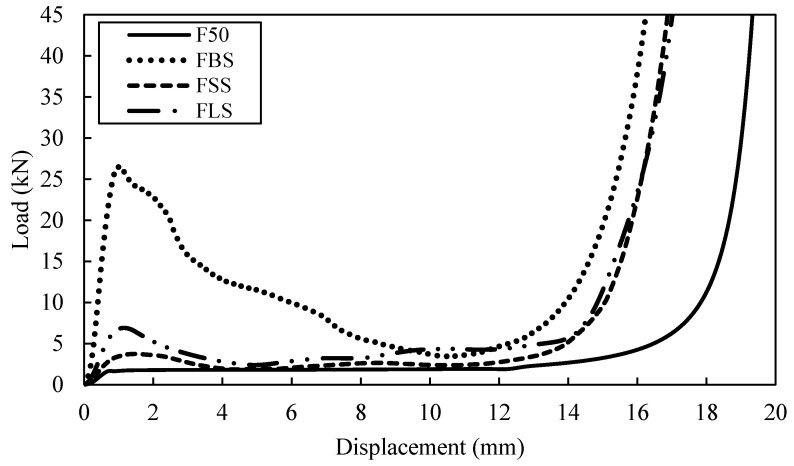
Load-displacement curve for F50 reinforced with different type of single tubes.

A series of tests are undertaken to characterize the energy-absorbing behaviour of the single-tube reinforced foam structure. Figure 7 shows a comparison of typical compressive load-displacement curves following tests on F50, FBS, FSS and FLS. The curves for all tube reinforced structures exhibited similar traits, which is linear response before the fracture occurred to the structure at approximately 1 mm. During this phenomenon, the stiffness for the tube reinforced foam structure will reduce. Non-linear response appears in the curve after the peak load is attained until the compression is in a stable mode with an approximately constant force, before declining throughout the last phase of the test. An examination of the curves for FBS structure indicates that the load increases linearly up to roughly 26.6 kN. This is followed by FLS and FSS which have the peak load at 6.9 kN and 4.5 kN respectively. The benchmark sample, which is F50, provide the lowest maximum load when it started to fracture compared to the other three types of tube reinforced foam structure.

**Figure 8:**
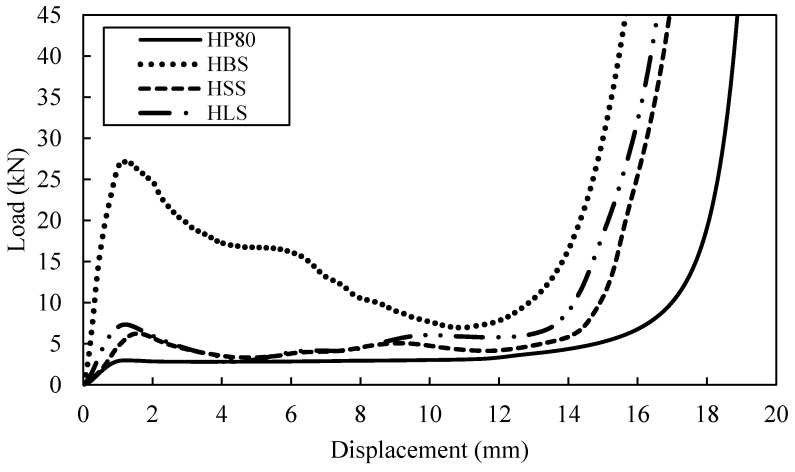
Load-displacement curve for HP80 reinforced with different type of single tubes.

A typical load-displacement curve for tube reinforced with HP80 foam is presented in Figure 8. The density of the foam used is 80 kg/m^3^ which is denser than the F50 foam. Here, the HBS structure load clearly rise up to 27 kN at 1.3 mm and drop gradually before the densification point at approximately 12.5 mm. Conversely, the curve for HSS and HLS exhibit similar traits under compression load. The initial fracture of HSS structure takes place when the load increases up to 6.3 kN while the HLS structure starts to fail at 7.3 kN which is slightly higher than HSS structure. The curve for three tube reinforced foam structures obviously showed that the strength of the reinforced structure is better than the benchmark sample, i.e., HP80. The increase of density foam in the tube reinforced foam structure leading to slightly changes in the strength of the structure. The increment of strength value for HBS and HLS structures are not more than 6% when compared to FBS and FLS structures. However, for the HSS structure, an increment of approximately 40% in strength is recorded.

Based on the energy absorption under the load-displacement curves for both F50 and HP80 foams, the bamboo reinforced foam structure offers an energy-absorbing capability greater than both PVC reinforced foam structures. The SEA values for FBS, FLS and FSS structures are found to be 17.9, 10.0 and 8.2 kJ/kg, respectively. In contrast, the SEA value for HBS, HLS and HSS are 24.5, 10.9 and 10.1 kJ/kg, respectively. Previous study by Umer et al. [15] has found that the SEA values is around 20 kJ/kg for single bamboo tube (D/t = 4.6) reinforced foam structure which is almost similar to the value for FBS and HBS. However, it is interesting to note that the SEA increases with the increasing density foam, similar to study conducted by Alia [1].

**Figure 9:**
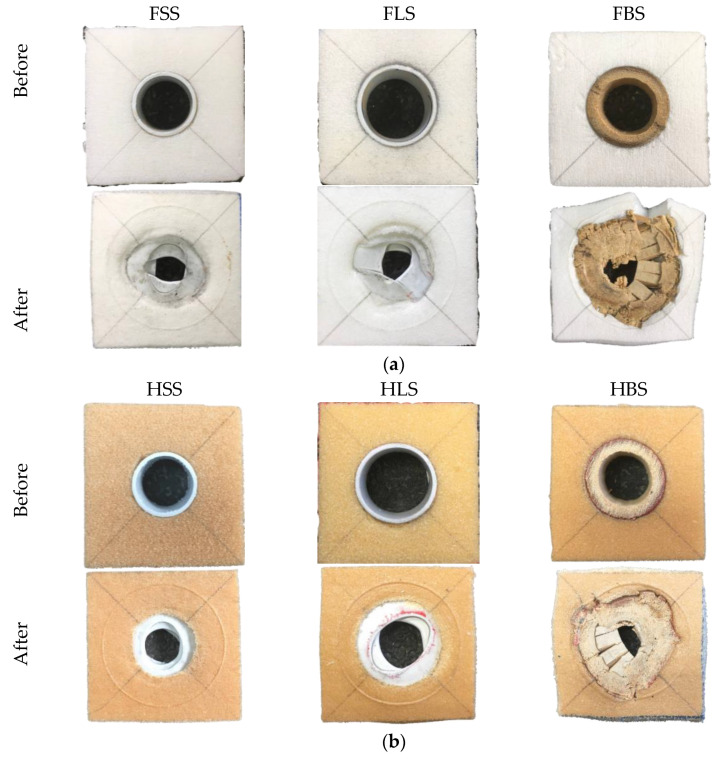
The top view of single tube reinforced with (**a**) F50, and (**b**) HP80 foam structures.

The deformation of the single tube reinforced foam structure after completing the compression process is shown in Figure 9. All the tubes were compacted and locked up at densification region when the tube walls have fully collapsed, as suggested by Rajput et al. [14]. Here, the lateral movement of tube reinforced structure is limited by increasing the foam density from 50 to 80 kg/m^3^. In Figure 9b, there is only a small amount of bamboo tube splitting is visible on the outside diameter. A closer look reveals that numerous of these fractures have penetrated to the neighbouring foam, similar to [15]. For PVC reinforced foam structures, the buckling and folding failure also being restricted with the increasing of foam density. Thus, the foam has been successful in constraining the lateral movement and failure mechanism.

### 3.4. Multi Tube Reinforced Foam Structures

The following part of this study focused into the effect of multiple tube reinforced foam structures on its energy-absorbing response. Here, four tubes are used and embedded into the foam similar to the single tube.

**Figure 10:**
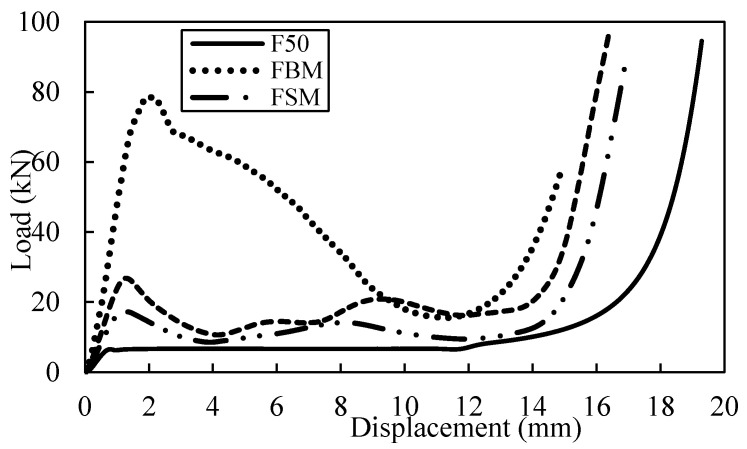
Load-displacement curve for multiple tube reinforced with F50 foam structure.

**Figure 11:**
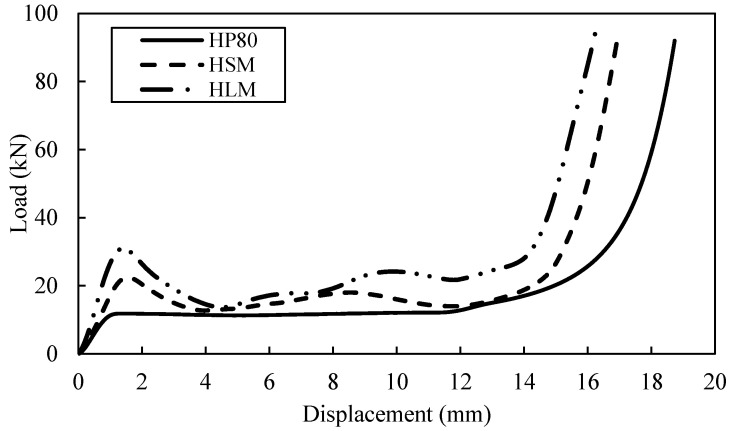
Load-displacement curve for multiple tube reinforced with HP80 foam structure.

In this study, the multiple tube reinforced with HP80 foam offer better energy absorption than F50 foam due to the higher density in HP80 as shown in Figure 10 and Figure 11. It is observed that the energy absorption for FBM (F50 foam) and HLM (HP80 foam) are the most efficient. Both structures offer greater area of load-displacement curves that is related to SEA value. An increase value of SEA is recorded from 17.9 kJ/kg to 20.9 kJ/kg for FBS and FBM structures (from single to multiple). Relatively, for HLS and HLM structures, the SEA value increase from 10.9 kJ/kg to 11.5 kJ/kg. However, the SEA values from single to multiple tube reinforced foam structure did not vary significantly due to the influence of the initial mass of the structure as it is inversely proportional to the SEA value, as proposed by Alantali et al. [16]. Invariably, the bamboo structures always dominated in any cases. However, the limitation for the testing machine used in this work is not capable of achieving compression loads exceeding 100 kN. This explained why the HBM specimen is not carry on being tested and included in Figure 11 as it has exceeded the machine ability.

**Figure 12:**
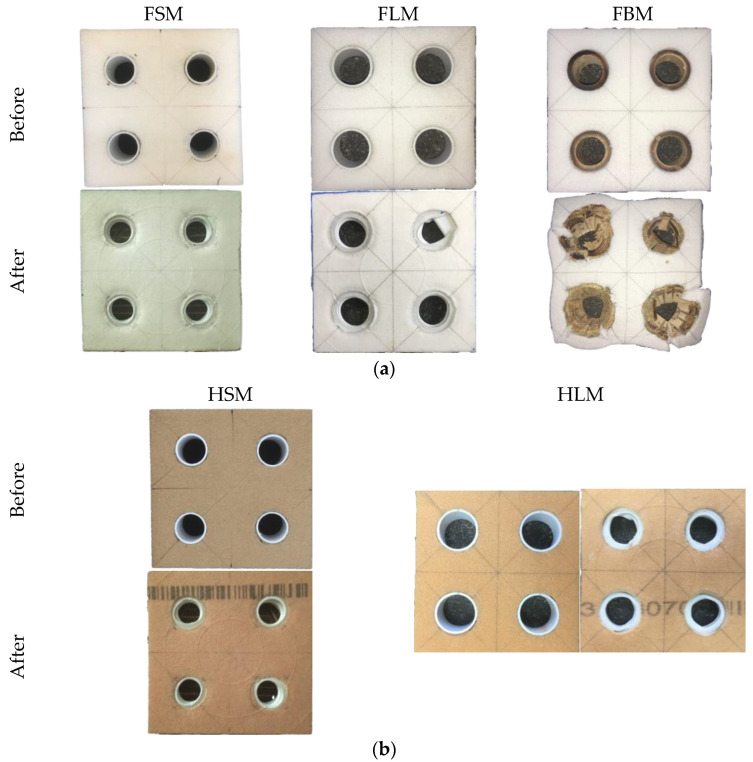
The top view of multiple tube structure with (**a**) F50 and (**b**) HP80 foam strucctures.

**Figure 13:**
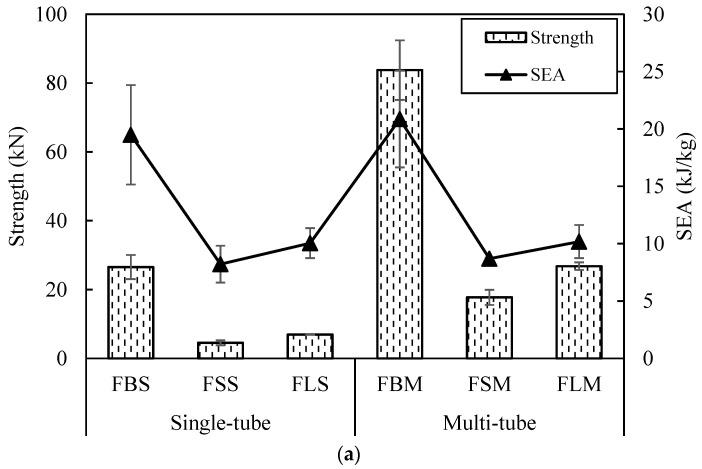

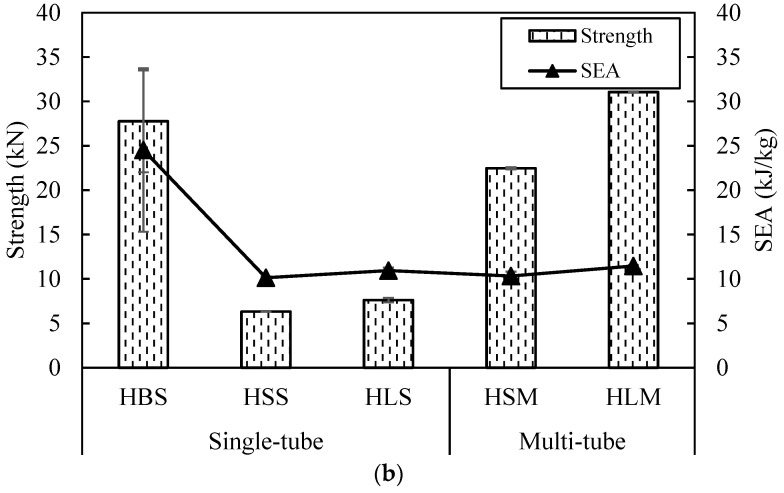
Compressive strength and SEA for single and multiple tube embedded in (**a**) F50 and (**b**) HP80 foams.

Furthermore, the SEA value and strength found to be increased when there is an increasing in tube diameter as well as the foam density. This shows that the local limitation imposed by the F50 and HP80 foam has a significant impact on the failure processes in tubes. In Figure 12, the deformation failure of multiple tube reinforced foam structures after testing is presented. The top section of the PVC tube shift slightly to one side when interpenetration occurred due to the transverse shear while the fracture of fibre occurred in the bamboo tube causes the tube to fractured into radial cracks and longitudinal splitting [7,17]. The failure of the bamboo tube can be seen clearly from the top view. As comparison to the Figure 9a, the failure of the single bamboo tube reinforced foam structure (FBS) is similar to the multiple bamboo tube reinforced foam structure (FBM) as shown in Figure 12a. The failed bamboo tube pushed the foam and leads to a crack in the foam structure. This means that the neighbouring tube properties can be affected as well. Figure 13 compares the single and multiple tubes reinforced foam structures.

### 3.5. Influence of Crosshead Speed on Multiple Tube Reinforced Foam Structures

**Figure 14:**
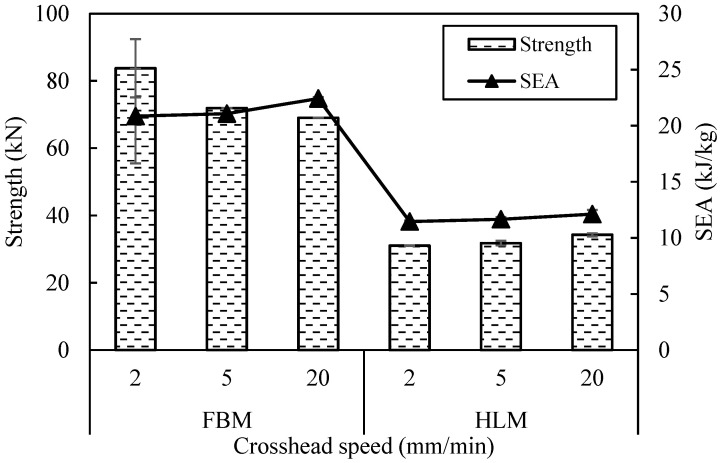
Compressive strength and SEA for FBM and HLM structure with three different compression crosshead speed.

Figure 14 presents the results for HLM structure under different crosshead speed displacement. At the initial stage, the peak force of the crosshead speed at 20 mm/min is only 3.3 kN higher than the 2 mm/min. This is maybe due to the crosshead speed used is not much in difference. However, the crosshead speed of 20 mm/min shows the highest peak force in comparison to the other, followed by 5 mm/min and lastly 2 mm/min. Here, as the crosshead speed is increasing, a higher value of compressive strength is obtained which is similar to the study by [18]. Additionally, by increasing the strain-rate will resulting a slight increase in the SEA capability of the reinforced foams, possibly due to the rate-sensitivity of the failure processes occurring in the reinforced structure.

## 4. Conclusions

The conclusions of this study can be drawn as follow:

1. Single bamboo tube reinforced foam structure offers highest value in specific energy absorption and compression strength. By increasing the foam density, it will increase the specific energy absorption and compressive strength value. A larger size of diameter of tube can enhance the specific energy absorption and compressive strength.
2. The compressive strength increases significantly when several tubes are introduced inside the foam. In contrast, the specific energy absorption does not change significantly for multiple tube reinforced foam structure. The multiple bamboo tube reinforced F50 foam structure offers better value of compression properties. In addition, the multiple PVC tube reinforced foam structure with a diameter of 25 mm gives the highest energy absorbing capability in HP80 foam.
3. The specific energy absorption does not vary significantly under the different crosshead speed below 20 mm/min. However, an increase in crosshead speed during testing can leads a slightly higher value of specific energy absorption. Therefore, the structures are believed to have a strain-rate effect when a higher speed is applied.

## Figures and Tables

**Table 1:** Physical properties of PVC and Bamboo tubes.

Tube	Outer Diameter, D_o_ (mm)	Inner Diameter, D_i_ (mm)	Thickness (mm)	D_i_/t Ratio
PVC 20	20	17	1.5	11.3
PVC 25	25	21	2.0	10.5
Bamboo	25	18	3.5	5.1

**Table 2:** Code used for the tube reinforced foam structures.

Type of Specimen		Code	
**Foam**	**Tube**	**Single**	**Multiple**
F50	Bamboo Ø 25 mm	FBS	FBM
F50	PVC Ø 20 mm	FSS	FSM
F50	PVC Ø 25 mm	FLS	FLM
HP80	Bamboo Ø 25 mm	HBS	HBM
HP80	PVC Ø 20 mm	HSS	HSM
HP80	PVC Ø 25 mm	HLS	HLM

**Table 3:** Compression properties of foam.

Foam	Density (kg/m^3^)	Compressive Load (kN)	Compressive Strength (MPa)	Compressive Modulus (MPa)	SEA (kJ/kg)
F50	50	1.76	0.70	26.45	7.89
HP80	80	2.99	1.20	29.66	9.46

## References

[1] Alia R.A. (2015). The Energy-Absorbing Characteristics of Novel Tube-Reinforced Sandwich Structures. Ph.D. Thesis.

[2] Cinar K. (2020). Evaluation of sandwich panels with composite tube-reinforced foam core under bending and flatwise compression. J. Sandw. Struct. Mater..

[3] Birman V., Kardomateas G.A. (2018). Review of current trends in research and applications of sandwich structures. Compos. Part B Eng..

[4] Colloca M., Dorogokupets G., Gupta N., Porfiri M. (2012). Mechanical properties and failure mechanisms of closed-cell PVC foams. Int. J. Crashworthiness.

[5] Shinde R.B., Mali K.D. (2018). An Overview on Impact Behaviour and Energy Absorption of Collapsible Metallic and Non-Metallic Energy Absorbers used in Automotive Applications. IOP Conf. Ser. Mater. Sci. Eng..

[6] Khan R.A., Mahdi E., Cabibihan J.J. (2021). Effect of fibre orientation on the quasi-static axial crushing behaviour of glass fibre reinforced polyvinyl chloride composite tubes. Materials.

[7] Zuhri M.Y.M., Liao Y., Wang Q.Y., Guan Z.W. (2019). The energy absorbing properties of bamboo-based structures. J. Sandw. Struct. Mater..

[8] Zhou J., Guan Z., Cantwell W.J. (2018). The energy-absorbing behaviour of composite tube-reinforced foams. Compos. Part B Eng..

[9] Alhawamdeh M., Alajarmeh O., Aravinthan T., Shelley T., Schubel P., Kemp M., Zeng X. (2021). Modelling hollow pultruded FRP profiles under axial compression: Local buckling and progressive failure. Compos. Struct..

[10] Alia R.A., Cantwell W.J., Langdon G.S., Yuen S.C.K., Nurick G.N. (2014). The energy-absorbing characteristics of composite tube-reinforced foam structures. Compos. Part B Eng..

[11] Molari L., Mentrasti L., Fabiani M. (2020). Mechanical characterization of five species of Italian bamboo. Structures.

[12] ASTM (2003). Standard Test Method for Flatwise Compressive Properties of Sandwich Cores 1. Am. Soc. Test. Mater..

[13] Li Q.M., Magkiriadis I., Harrigan J.J. (2006). Compressive strain at the onset of densification of cellular solids. J. Cell. Plast..

[14] Rajput M.S., Burman M., Köll J., Hallström S. (2019). Compression of structural foam materials—Experimental and numerical assessment of test procedure and specimen size effects. J. Sandw. Struct. Mater..

[15] Umer R., Balawi S., Raja P., Cantwell W.J. (2014). The energy-absorbing characteristics of polymer foams reinforced with bamboo tubes. J. Sandw. Struct. Mater..

[16] Alantali A., Alia R.A., Umer R., Cantwell W.J. (2019). Energy absorption in aluminium honeycomb cores reinforced with carbon fibre reinforced plastic tubes. J. Sandw. Struct. Mater..

[17] Wei Y., Zhou M., Zhao K., Zhao K., Li G. (2020). Stress–strain relationship model of glulam bamboo under axial loading. Adv. Compos. Lett..

[18] Xiang C., Xiao Z., Ding H., Wang Z. (2020). Compressive Properties and Energy Absorption Characteristics of Extruded Mg-Al-Ca-Mn Alloy at Various High Strain Rates. Materials.

